# EBS in Children with De Novo Pathogenic Variants Disturbing *Krt14*

**DOI:** 10.3390/ijms25052989

**Published:** 2024-03-04

**Authors:** Anastasiya V. Kosykh, Irina I. Ryumina, Alexandra S. Botkina, Nadezhda A. Evtushenko, Elena B. Zhigmitova, Aleksandra A. Martynova, Nadya G. Gurskaya, Denis V. Rebrikov

**Affiliations:** 1 Center for Precision Genome Editing and Genetic Technologies for Biomedicine, Pirogov Russian National Research Medical University, Ostrovityanova 1, Moscow 117997, Russia; avkosyh@gmail.com (A.V.K.); lenae7777@gmail.com (E.B.Z.); aleksandramartynova077@gmail.com (A.A.M.);; 2 National Medical Research Center for Obstetrics, Gynecology and Perinatology named after Academician V. I. Kulakov, ul Akademika Oparina, 4, Moscow 117997, Russia; 3 Department of Dermatovenereology, Russian Children’s Clinical Hospital, Pirogov Russian National Research Medical University, Leninsky Prospekt, 117, k3, Moscow 119571, Russia

**Keywords:** epidermolysis bullosa simplex, epidermis, genodermatoses, keratin 14, de novo mutation

## Abstract

Epidermolysis bullosa simplex (EBS) is a dermatological condition marked by skin fragility and blister formation resulting from separation within the basal layer of the epidermis, which can be attributed to various genetic etiologies. This study presents three pathogenic de novo variants in young children, with clinical manifestations appearing as early as the neonatal period. The variants contribute to the EBS phenotype through two distinct mechanisms: direct keratin abnormalities due to pathogenic variants in the *Krt14* gene, and indirect effects via pathogenic mutation in the *KLHL24* gene, which interfere with the natural proteasome-mediated degradation pathway of KRT14. We report one severe case of EBS with mottled pigmentation arising from the Met119Thr pathogenic variant in KRT14, another case involving a pathogenic KLHL24 Met1Val variant, and a third case featuring the hot spot mutation Arg125His in KRT14, all manifesting within the first few weeks of life. This research underscores the complexity of genetic influences in EBS and highlights the importance of early genetic screening for accurate diagnosis and management.

## 1. Introduction

Epidermolysis bullosa simplex (EBS) is characterized by fragility and the blistering of skin due to a cleavage within the epidermal basal layer, stemming from diverse genetic causes. Around three-quarters of individuals who are clinically identified as having EBS carry genetic alterations in the *Krt5* or *Krt14* genes. These genes code for keratin proteins that are crucial for the integrity and resilience of the skin. A particularly aggressive form of EBS is often associated with mutations that occur in the highly conserved regions of the keratin’s central rod domain. These regions include the helix initiation peptide and helix termination peptide motifs, which are critical for the proper assembly of keratin filaments. Mutations in these areas, especially within the 1A domain of the keratin protein, are believed to disrupt the formation of keratin heterodimers, leading to weakened structural stability in skin cells and resulting in the characteristic blistering seen in EBS.

Three pathogenic de novo variants are described in young children, starting from the neonatal period of age. Three variants lead to the clinical symptoms of EBS either by directly causing abnormalities in keratin (pathogenic mutations in the *Krt14* gene) or indirectly (pathogenic mutations in the *KLHL24* gene) by disrupting natural proteasome-mediated degradation of KRT14.

## 2. Case Description

### 2.1. EBS Severe with Mottled Pigmentation (EBS-MP)

Proband A is the second child in a family with healthy parents and healthy sibling. Signs of a severe form of EBS, formerly referred to as Dowling–Mear, were observed from the first days of life. At birth, multiple extensive blisters with an erosive surface opening were observed on the skin of the hands and feet; on the left foot, there was bleeding erosion. In addition, the skin was macerated, and hyperkeratosis of soles and palms was observed. The patient’s most difficult period, in terms of the severity of disease manifestations, was the 1st year of life. Over time, a gradual decrease in the frequency of rashes and blister size was observed, although exacerbations still occurred in spring and summer. Perinatal pathology of the central nervous system—central nervous system depression syndrome—was additionally diagnosed.

Beginning at 9 months old, initial areas of hyperpigmentation emerged and became permanent at the locations where previous inflammation and blistering had occurred on the wrist and hand. This pigmentation manifested on the leg a few months later. At the same age, complications with the digestive system, specifically in the epigastric region and intestines, were first observed.

The child demonstrated strongly delayed development, began to walk after 2 years of age. At this age, after a neurological examination regarding freezing, EPI activity was detected on the EEG and, finally, somatic focal epilepsy was diagnosed. MR findings were consistent with minimal periventricular leukopathy, and weak expansion of the lateral ventricles of the brain was also observed. At the age of 2, a genetic study was carried out and the *Krt14* mutation was revealed.

**Figure 1:**
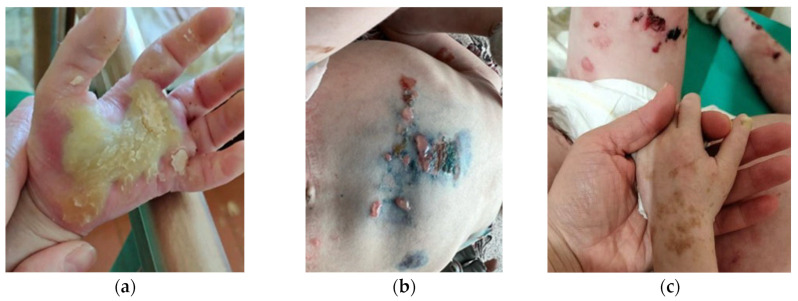
Clinical image of Proband A with KRT14 Met119Thr at the age of 5. (**a**) Palmoplantar hyperkeratosis; (**b**) Skin blisters on the abdomen: erosions heal without scarring; (**c**) Areas of mottled pigmentation were observed on limbs: pigmented zones arise in the areas of blisters after healing.

An extensive clinical examination was continued on patient A at the age of 5 in the Dermatovenerology Department of the Russian Children’s Clinical Hospital. At this age, Proband A still spoke with difficulty. The pathological skin condition was characterized as a subacute inflammatory process that is generalized and asymmetrical. It predominantly affects the skin on the face, neck, torso, and both the upper and lower extremities, including involvement of the palms and soles (Figure 1a,b). Clinically, it presents with numerous blisters on smooth skin areas, containing serous or serosanguinous fluid. These blisters are arranged in garland-like patterns or clusters, reminiscent of a herpetiform distribution. Additionally, the skin exhibits multiple erosions encircled by remnants of blistered skin, along with serous and serosanguinous crusts. There are also atrophic and normotrophic scars marking the locations of previously resolved bullous eruptions.

On the skin of the forearms, hands, and thighs, one can observe multiple hyperpigmented macules with irregular shapes and light brown hues (Figure 1c). These macules have distinct, uneven, and occasionally serrated margins. Dermatoscopy reveals non-melanocytic lesions, which are attributable to an excessive accumulation of melanin within the keratinocytes.

The palms and soles exhibit signs of hyperkeratosis and deep linear fissures. The skin surrounding the lesions appears thinned but retains normal elasticity and turgor. The nail plates are dull and have a yellowish discoloration. Onychogryphosis is marked and onycholysis is evident in some areas. The hair is brittle, dry, and exhibits slow growth. Within the oral cavity, there is a solitary erosion on the tongue, which is coated with a thin layer of fibrin. Other mucous membranes appear unaffected.

**Figure 2:**
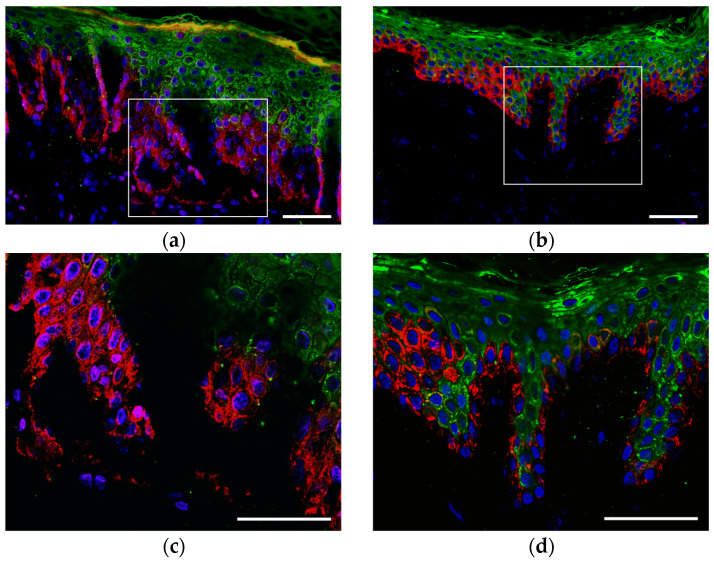
Immunohistochemistry (IHC) of the skin cryosections from (**a**,**c**) Proband A and (**b**,**d**) healthy control. Skin sections were stained for KRT10 (green) and KRT14 (red). Nuclei were stained with DAPI (blue). Scale bar—50 μm. The rectangle on low (200×) magnification image (**a**,**b**) indicates the area captured on high (400×) magnification and demonstrates the area of the cleavage of the epidermis (**c**) compared with normal skin (**d**).

We received a punch biopsy from patient Proband A. The biopsy was obtained from tissue of the upper limbs in a crusted area following the formation of a blister with serous fluid. Histology shows an area of superficial skin cleavage above the basal keratinocytes in combination with a hypertrophied layer of the stratum corneum (Figure 2a,c). The histology of the skin biopsy from a healthy donor with normal morphology is shown for comparison (Figure 2b,d).

**Figure 3:**
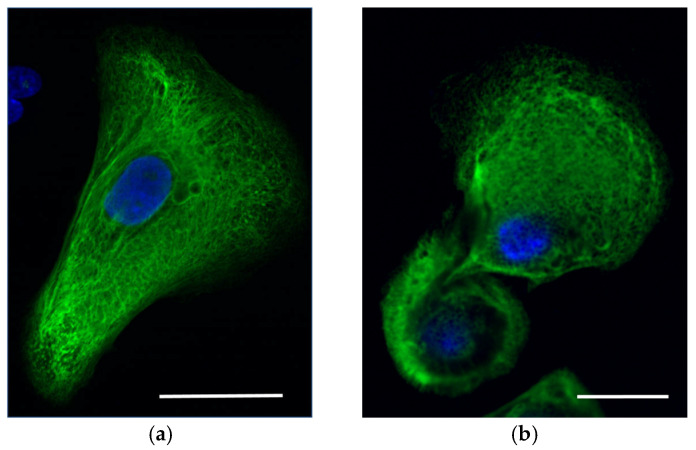
Immunocytochemistry of epidermal keratinocytes in vitro from (**a**) Proband A and (**b**) healthy control. Cells were stained for KRT14 (green). Nuclei were stained with DAPI (blue). Scale bar—20 μm.

Immunocytochemistry analysis with antibodies specific to KRT5/14 did not reveal massive tonofilament clumps in the keratinocytes. However, a mild trend was possibly observed in the increase of aggregate formation of the keratin network in comparison with the keratinocytes of the healthy donor (Staining against KRT14, Figure 3).

Results of next-generation sequencing (NGS) revealed heterozygous pathogenic variant in *Krt14* rs28928893 that have the effect of missense mutation c.356T>C (p.Met119Thr) (Table 1) previously described in 2006 [1,2]. This pathogenic variant was registered in HGVS:NC_000017.11:g.41586479A>G, NG_008624.1:g.5417T>C, belongs to OMIM #131760. NGS analysis of the parents and sibling of the Proband A did not show the presence of this mutation or any other possible pathogenic variants, though their sequences of *Krt14* possessed several common SNPs with Proband A. Sanger sequencing further confirmed the results of NGS.

### 2.2. Epidermolysis Bullosa Simplex, Generalized Intermediate, with or without Cardiomyopathy 

**Figure 4:**
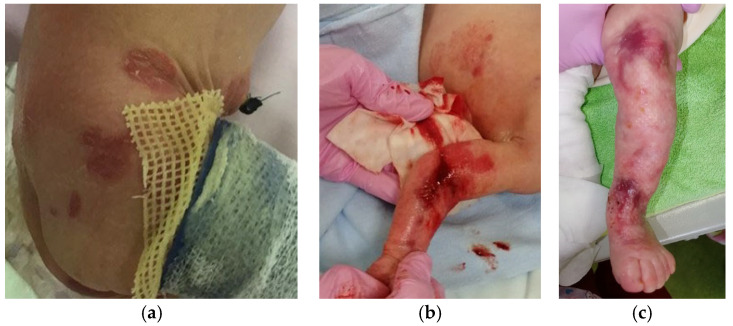
Prominent skin erosions of Proband B with KLHL24 Met1Val. (**a**) Body erosions on the first day of life; (**b**) New deep erosions on the third day of life; (**c**) Wound epithelialization on the twenty-sixth day of life. The patient received daily treatment with alcohol-free antiseptic solutions and the application of emollient and epithelializing topical preparations containing dexpanthenol to enhance skin healing. The affected skin areas were protected with a specialized mesh dressing, while sponge pads and soft, nonwoven cloths, along with gentle tubular bandages, were utilized for coverage.

Proband B, the second child in a family with no history of EBS, has a healthy older sister. From birth, the child exhibited significant skin abnormalities, including deep erosions ranging in size from 0.5 × 0.5 cm to 1.5 × 2.0 cm located on the legs, around the knee joints, on both the outer and inner thighs, inside the elbow creases, on the backs of both hands, and on the sacrum (Figure 4). 

In addition, there were clusters of intact blisters measuring from 0.3 × 0.2 cm to 2.0 × 3.0 cm against a non-hyperemic background in the periumbilical region, on the anterior abdominal wall, and in the nasal area. By the second day of life, new blisters and erosive lesions had appeared on the right forearm and sacrum. Throughout the follow-up period, there was a periodic appearance of new blisters ranging from 0.2–0.3 mm to 6 cm in diameter, predominantly on the extremities. During the hospital stay, the child underwent a thorough examination. Neurosonography identified a first-grade intraventricular hemorrhage on both sides, which later resolved on its own. No additional pathological findings related to internal organs and systems were observed.

Whole exome sequencing of DNA isolated from peripheral blood cells revealed the variant rs886037956 c.1A>G in *KLHL24* gene in Proband B (Table 1). Since there was no family history of disease associated with genodermatoses and no mutation was found in the DNA of parents, the *KLHL24* variant found in Proband B was considered to be de novo mutation. The variant rs886037956 c.1A>G presents in a heterozygous state, which leads to the substitution p.Met1Val, the loss of the start codon and the synthesis of a protein of shorter length. This mutation is described in databases as associated with autosomal dominant epidermolysis bullosa simplex, intermediate-generalized, with scarring and hair loss (OMIM 617294).

Additionally, substitutions were detected in the *POGLUT1* gene rs587777297 c.835C>T in the heterozygous state and in the *PDE11A* gene rs76308115 c.169C>T in the heterozygous state (Table 1). Similar mutations in the heterozygous state were found in the healthy child’s father. Apparently, these substitutions are not the main cause of the disease of the Proband, but possibly contribute to, and modify, the clinical picture.

The mutant of *KLHL24* with a deleted former start codon had an altered N-terminal fragment structure, resulting in reduced ubiquitination and an extended lifetime of this ubiquitin ligase receptor protein. As a result, the mutant *KLHL24*—rs886037956 is a gain-of-function mutant KLHL24-ΔN28 that mediates the rapid ubiquitination and degradation of several proteins like keratin 15, desmin, and keratin 14. In patients with the same mutations, the clinical manifestations include alopecia, cardiomyopathy and epidermolysis bullosa syndrome [3].

Biopsies for histology and cell culture analysis in vitro were not obtained from Proband B for ethical reasons—the newborn was in the acute phase of the disease and surgical intervention would have led to the emergence of new inflammatory foci.

Among the most significant pathogenic variants found in Proband B is *POGLUT1* rs587777297 c.835C>T (p.Arg279Trp). This sequence change replaces arginine, which is basic and polar, with tryptophan, which is neutral and slightly polar at codon 279 of the POGLUT1 protein (p.Arg279Trp). This variant is present in population databases (rs587777297, gnomAD 0.006%). Interestingly, this missense change has been observed in individual(s) with Dowling-Degos disease [4]. Variation ID: 126532 in ClinVar contains an entry for this variant. Numerous in silico predictions, based on the advanced modeling of protein sequence and comparison of structural and functional properties with spatial information of the amino acid residues demonstrated that this missense variant of POGLUT1 protein has impaired protein function. Then, the disruption of POGLUT1 missense function was confirmed in experimental studies [5].

### 2.3. EBS Severe with Recurrent Arg125His Krt14 Variant

**Figure 5:**
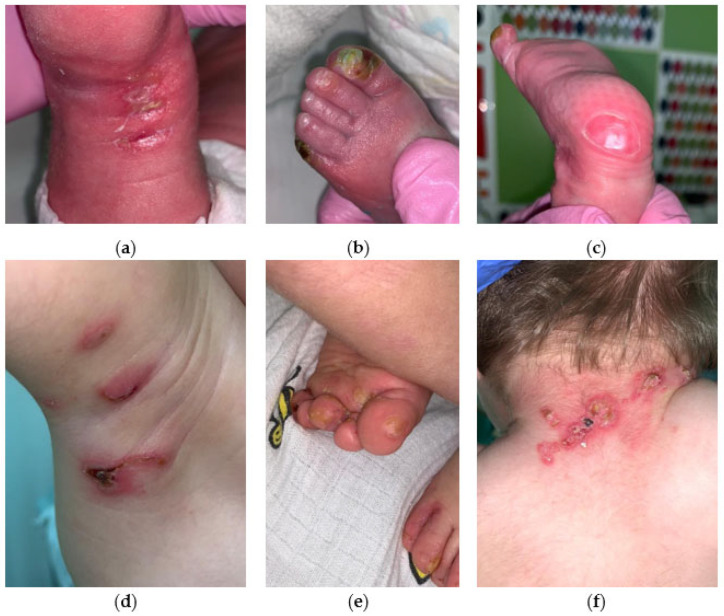
Clinical image of Proband C with KRT14 Arg125His on (**a**–**c**) the 18th day of life and (**d**–**e**) at 1 year of age. From the moment of birth and during the observation period, the new erosions (**a**,**d**), areas with hyperkeratosis (**b**,**e**) and blister (**c**,**f**) formations were registered.

EBS severe with hotspot mutation in *Krt14* converting arginine at position 125 to histidine. Patient C has recurrent pathogenic variant KRT14 Arg125His. The boy was born prematurely (36 weeks) from a dichorionic diamniotic twin pregnancy after in vitro fertilization. The general condition at birth was of average severity due to signs of morpho-functional immaturity and thermolability. Dryness and skin cracks have been noted since birth. The condition included negative dynamics on the 5th day due to the appearance of blisters with transparent content, without hyperemia, ranging in size from 1.0 × 0.5 to 1.5 × 1.0 cm on the skin of the toes, dorsal side of the foot, and in places of cracks (Figure 5a–c).

There was periodic appearance of new rashes in the form of blisters in diameter from 0.2–0.3 mm to 1.5 × 0.7 cm on the upper and lower extremities and oral mucosa. The skin of the neck exhibited multiple erosions, surrounded by serous and serous-hemorrhagic crusts. Additionally, bullous rashes were observed on the skin of the upper and lower extremities of the child (Figure 5d,e). The development of an infectious process, including herpetic infection, was excluded. The severe form of EBS was diagnosed first and then confirmed by molecular genetic study. High-throughput exome sequencing was performed and followed by searching for pathogenic or probably pathogenic variants with uncertain significance in genes associated with epidermolysis bullosa and other skin lesions. The following recurrent missense mutation in the *Krt14* gene was found: variant rs58330629 in heterozygous state in exon 1 of *Krt14* (Table 1). This variant leads to amino acid substitution of p.Arg125His in KRT14. As no skin lesions were found in the second child of the twin, as well as in their parents, presumably this case represents de novo recurrent mutation, although the confirmation of de novo state is still needed. Unlike the case with the same mutation described by Shemanko [6], Proband C has no hoarse cry.

## 3. Discussion

### 3.1. The Pathogenic Variants Induced Severe and Intermediate Type of EBS

Approximately 75% of patients clinically diagnosed with epidermolysis bullosa simplex have been found to possess mutations in either the *Krt5* or *Krt14* genes [7]. It has been shown that a particularly severe course of EBS occurs when mutations localize to highly conserved boundaries of the central rod domain of keratins, specifically in the helix initiation peptide and helix termination peptide motifs. The mutations located within the 1A domain of the keratin molecule are predicted to impair the structure of heterodimeric keratin molecules [8]. 

Disruption to the structure of the molecule leads to dysregulation of keratin assembly and organization, which could be evidenced by the formation of keratin aggregates. These result in altered turnover of the mutant protein, thereby contributing to the pro-inflammatory clinical manifestations observed in the epidermis of patients with EBS. 

Direct correlation between the localization of mutations in the *Krt5* and *Krt14* domains and the corresponding EBS subtype has been established and postulated [9,10]. Amino acid residues of highly conserved helix boundary motifs (helix initiative of terminative motif) are supposed to be more important for keratin stability than those located more centrally in a rod domain.

All described here mutations of *Krt14* and *KLHL24* are dominant negative with an autosomal dominant pattern of inheritance, as the most pathogenic variants of EBS. 

Two variants described here are located in 1A domain of KRT14, in helix initiation motif (HIP). One of these variants, Arg125His, represents a hotspot mutation in EBS [11,12]. The recurrent nature of the KRT14 Arg125 mutation and its high rate of spontaneous occurrence underscore the significance of these specific nucleotides in the pathology of EBS. It is hypothesized that this hotspot mutation may be linked to increased methylation and deamination at the local CpG site [10]. Helix initiation peptides are organized in a heptad structure specific to alpha–helical rod domains of keratin heterohybrids. Arg125 of KRT14 has the position ”g” in the HIP, and takes part in direct interaction with the “e” position of KRT5 [10]. Met119 of KRT14 has the “a” position of HIP, which belongs to another tight bond binding with “d“ of KRT5. Mutations affecting these interactions seem to be regularly associated with severe courses of EBS [13].

With the accumulation of data on mutations and the corresponding clinical picture in patients with EBS, the EB classification undergoes some changes over time. The most common EBS subtypes are localized (known as Weber–Cockayne), severe (known as generalized severe or Dowling–Meara (DM-EBS)) and intermediate (known as generalized intermediate or Köbner) EBS. Patients were diagnosed with DM-EBS when at least one of the following signs was present: 1. severe generalized herpetiform blisters, or 2. accumulation of tonofilaments in (supra-) basal cells visible in EM [7]. Proband A has severe herpetiform blisters, which corresponds to the first criteria for diagnosing DM-EBS. In addition, clinical manifestations of Proband A are similar to the Proband described by Cummins with identical mutation and severe DM-EBS diagnosis [14].

### 3.2. Severe EBS-MP

The vast majority of EBS with mottled pigmentation (EBS-MP) cases are caused by a mutation in *Krt5* gene, Pro25Leu. However, the cases of EBS-MP due to a mutation in KRT14 are also described, connected with only one pathogenic variant.

The pathogenic variant Met119Thr impacts the conserved initiation region of the first helical domain 1A of KRT14. This variant is situated within the highly conserved boundary initiative motif of the alpha–helix in keratin, leading to a marked disruption in protein folding. Notably, other genetic variants at this position, such as Met119Val and Met119Ile, result in less severe forms of EBS. This could be due to the retention of a nonpolar amino acid residue at position 119—methionine, valine, and isoleucine are all nonpolar—which is crucial for maintaining the structural integrity and functionality of keratin 14 [14]. The variant Met119Thr has proved to be de novo mutation, as the results of exome sequencing from the Quartet DNA reference materials (including the father, mother and two children of the family).

Electron microscopy data are unavailable in the case of our Probands, but according to Harrel et al., intraepidermal blister formation was found to be associated with tonofilament clumping in the case of the Proband with Met119Thr. Moreover, the hyperpigmentary zone of the skin biopsy confirmed increased melanin staining in the basal layer of the epidermis and the absence of dermal melanophages [2]. In the case of Proband A, no ultrastructural abnormalities of melanin granules were observed. Histological assessment of a pigmentary nevus showed findings typical of a benign compound melanocytic nevus.

Investigation of EBS cases with mottled pigmentation helps to clarify the relationship between keratinocytes, which control melanocytes’ mobility and adhesion; particularly, melanocyte adhesion in lesional skin. Melanocytes secrete special melanin-containing granules (melanosomes) to adjacent keratinocytes, mostly in the basal layer of epidermis. The differences in pigmentation consist mostly of variation in the number and size of melanosomes, as well as their composition and distribution, independent of the initial numbers of the melanocytes [15]. The close relationship between inflammatory skin conditions and abnormal pigmentation development is of deep interest, as it could bring about new possible therapeutic approaches [16]. The proposed mechanism of post-inflammatory hyperpigmentation includes the secretion of keratinocyte-derived soluble factors: proinflammatory prostaglandin E2 and F2 alpha (PGE2, PGF2α), α-melanocyte-stimulating hormone (α-MSH), endothelin 1 (ET-1), stem cell factor (SCF), and hepatocyte growth factors (HGF), which bind to specific receptors, located on the melanocyte cell membrane, and induce the proliferation and differentiation of melanoblasts into melanocytes. The proinflammatory profile of EBS keratinocytes includes the secretion of IL-1B and IL-6 and stimulation of the indirect production of melanin. Other keratinocytes specific interleukins like IL-33 and GM-CSF are responsible for the upregulation of tyrosinase-related proteins 1 and 2 (TYRP 1 and 2) via the activation of MAPK or PKA pathways, and IL-18 directly upregulates tyrosinase activity, which is important in the process of melanin production.

Almost no aggregates were detected in the primary keratinocytes of Proband A by immunocytochemistry analysis despite the severity of the clinical manifestations of EBS. However, the small number of aggregates are seen at the periphery of the cell (Figure 3a).

Similar clinical features were demonstrated earlier for two patients with missense Met119Thr in KRT14, such as persistent, severe keratoderma, thick pachyonychia, impressive nail thickening and onychogryphosis, the unusual early onset of severe hyperkeratosis [1]. Moreover, similar signs of underdevelopment were described, as the patients are still not walking at the age of 2.

### 3.3. Intermediate EBS with KLHL24 Mutation

Proband B has *KLHL24* start-codon mutation (Met1Val) that leads to a truncated protein, with Met29 being the new start mutant protein KLHL24-ΔN28, which mediates accelerated KRT14 degradation in cells. KLHL24 is a part of ubiquitin ligase that participates in the process of proteasome degradation. Due to the positive feedback mechanism, KLHL24 itself serves as a template for ubiquitination, which means a rather short lifetime of this protein in the cell. The pathogenic variant rs886037956 was first described in 2016 [3,17]. Moreover, VarSome connects this variant with EBS patients who are also diagnosed with dilated cardiomyopathy (DCM) [18]. The mechanism of such DCM pathology development was proposed, by which desmin, as the cardiac homologue of KRT14, is subjected to excessive degradation by mutant form of ubiquitin ligase, KLHL24-ΔN28. Indeed, isogenic cardiomyocytes differentiated from patient-specific induced pluripotent stem cell line exhibit tenfold diminished levels of desmin expression.

Two other pathogenic variants found in Proband B were rs76308115 in *PDE11A4* and rs587777297 in *POGLUT1*, respectively. 

The *PDE11A4* gene is expressed in the adrenal cortex and encodes enzyme phosphodiesterase-11A4. PDE11A4 catalyzes the hydrolysis of cyclic adenosine monophosphate and cyclic guanosine monophosphate. The variant rs76308115 is pathogenic, according to ClinVar (VCV000005286.14); the pathology connected with this variant is adrenocortical disease, in which numerous pigmented micronodules develop in the adrenal glands [19]. The pathogenic variant NM_016953.4:c.919C>T induces a nonsense codon instead of 307Arg shown in individuals with adrenocortical hyperplasia [20]. No obvious connection of this condition has been found in Proband B with EBS clinical manifestation.

Interestingly, another pathogenic variant found in Proband B—*POGLUT1* rs587777297 c.835C>T (p.Arg279Trp) was shown previously in patients with genodermathosis, Dowling-Degos disease 4 (DDD4) or Galli-Galli [4,21]. This autosomal-dominant genodermathosis is marked by the gradual development of a net-like pattern of darkened skin areas that may be considered cosmetically unappealing or damaging [22]. *POGLUT1* encodes the enzyme O-glucosyltransferase 1, which plays an active role in the processes of Notch signaling. Functionally, POGLUT1 transfers O-linked glucose from UDP-glucose to serine residues in Notch EGF repeats with the consensus C1-X-S-X-P-C [23]. The enzymatic activity of POGLUT1 is known to be key to its regulatory role in the Notch pathway, as post-translational O-glycosylation of NOTCH1 intracellular domain influences cell proliferation. Abnormal pigmentation of skin and hair as a consequence of Notch deficiencies has been previously noted [24]. Dowling-Degos was previously attributed to the loss-of-function mutations in *Krt5*, and was found to be caused by mutations in the *POGLUT1* gene [21]. Later, other findings supported the role of pathogenic variants of POGLUT1 in DDD4 development [25,26]. Considering the clinical manifestations of Proband B, we have not yet noticed any signs of DDD4, although the age of onset of disease was reported to be between the second and sixth decade of life [21].

### 3.4. EBS Severe with Recurrent Arg125His 

Position 125 on the protein is a common site for mutations; namely, hotspot mutation, with various substitutions of the arginine at this position (such as serine, glycine, cysteine, proline, and leucine) commonly observed in patients with epidermolysis bullosa simplex. Interestingly, there were cases of severe, intermediate and localized forms of EBS with Arg125Cys, Arg125His and Arg125Gly mutations [13].

All three mutations reported here are de novo mutations causing severe EBS, as none of these pathogenic variants were found in the parents’ peripheral blood cell DNA, and/or there was no history of blistering diseases in the respected family. Seemingly, a high rate of *de novo* mutations was observed previously in the cohort of patients with EBS [27].

## 4. Material and Methods

### 4.1. Keratinocytes Culture Obtaining

The skin biopsy sample was incubated in a 0.2% Dispase solution (Life Technologies, Bleiswijk, The Netherlands) overnight at +40C. The next day, the epidermis layer was separated from the dermis and washed in a PBS solution without Ca+ and Mg+. The epidermis was incubated in a 0.05% Trypsin-EDTA solution (Life Technologies, Bleiswijk,The Netherlands) at 370C for 15–20 min, and then the trypsin solution was inhibited with FBS. The sample was centrifuged and the keratinocytes were resuspended in Cnt07 medium (CELLnTEC, Bern, Switzerland) and seeded into a culture dish with a collagen substrate.

### 4.2. Obtaining DNA from Human Peripheral Blood Mononuclear Cells (hPBMC)

Venous blood samples of all subjects were collected in EDTA-containing tubes. A standard technique was used for the isolation of hPBMC, which includes density centrifugation with the Ficoll–Paque gradient, as described in Grievink’s work [28]. Next, the ExtractDNA Blood and Cells kit (Evrogen, Moscow, Russian Federation) was used to isolate total DNA from whole blood and cells of animal origin in accordance with the manufacturer’s recommendations.

### 4.3. Exome Sequencing and Data Analysis

The patient’s DNA was analyzed on an IlluminaNextSeq 500 next-generation sequencer using the paired-end read method (2 × 75 bp). For sample preparation, a technique for selective capture of DNA regions belonging to the coding regions of more than 20,000 genes (Illumina TruSeq^®^ ExomeKit and IDT xGen^®^ Exome Research Panel) was used. The nomenclature provided at http://varnomen.hgvs.org/recommendations/DNA version 2.15.11 (accessed on 10 March 2017) was used to name the identified variants. Sequencing data were processed using a standard automated algorithm offered by Illumina for data analysis provided at https://basespace.illumina.com (accessed on 18 December 2017). Samples from the 1000 Genomes Project, ESP6500 and The Genome Aggregation Database were used to estimate population frequencies of identified variants. The OMIM database, the HGMD^®^ Professional version 2019.1 pathogenic variants database, specialized disease-specific databases (where available), and literature data were used to assess the clinical relevance of the identified variants. The total number of reads is 39.660.477 with 39.534 different variants. An average coverage is 49.9 with 3.2 as the number of fragments (%) with coverage less than ×10 (for WES). Only one variant was found after filtering by basic pathogenicity criteria and evaluation by clinical criteria: chr17:39742731A>G, c.356T>C in *Krt14* gene. Only variants with possible relevance to the patient’s clinical manifestations are included in the conclusion.

Sequencing of protein-coding human genes by paired-end reads was performed using targeted enrichment of genomic DNA. Illumina NovaSeq 6000 was used for the exome sequencing of the sample Proband C. The sequencing data were analyzed using an automated algorithm that includes evaluation of sequencing quality parameters (FASTQC module); removal of adapters and sequences with low quality (SEQPURGE module); alignment of reads to the hg19 version of the human genome (BWA MEM module); filtering of optical and PCR duplicates (SAMBLASTER module); local optimization of alignments (ABRA2 module); variant detection and filtering according to quality (FREEBAYES package); and annotation of variants relative to databases with clinical information (ENSEMBL-VEP module).

### 4.4. Immunohistochemistry of KRT14 in Skin Sections

The skin biopsies were fixed in 10% neutral buffered formalin at room temperature (RT) for 24 h. Fixed samples were washed three times in PBS at RT for 30 min each, followed by 12 h incubation in 30% sucrose solution at RT. Samples were frozen using liquid nitrogen in Tissue-Tek OCT Compound (Sacura, Hatfield, PA, USA) and sliced to 5 μm thickness on Thermo Scientific Microm HM 525 Cryostat. The skin slices were fixed with 10% formalin at RT for 30 min followed by three 5 min PBS washes, and blocked with 2.5% BSA (Merck Life Science LLC, Moscow, Russian Federation) in PBS at RT for 30 min. The primary antibodies were diluted in the blocking solution (PBS, 0.1% Triton X-100, 0.5% Tween, 1% serum, NAN3)—1:100 dilution for anti-KRT10 (Invitrogen, #MA1-06319) and 1:1000 dilution for anti-KRT14 (Abcam, #ab181595) and incubated overnight at 4 °C. Then, the slices were rinsed three times with PBS for 10 min each, followed by incubation with the secondary antibodies with Alexa Fluor 488-conjugated and Alexa Fluor 546-conjugated, respectively, diluted 1:750 in the blocking solution. After the 2 h RT incubation, the secondary antibody was removed and the slices were rinsed three times with PBS for 30 min each. The nuclei were stained for 20 min at RT with DAPI diluted 1:1000 in PBS. Finally, the slices were mounted with 20% glycerol and coverslipped.

### 4.5. Confocal Imaging

Confocal images were made using a LSM 880 confocal scanning microscope (Carl Zeiss Microscopy GmbH, Jena, Germany) based on the inverted fluorescent microscope Axio Observer.Z1 Zeiss equipped with six laser lines (633, 594, 561, 543, 514, 488 and 405 nm), five objectives (EC Plan-Neofluar 5×/0.16, EC Plan-Neofluar 10×/0.3, PL APO 20×/0.8, PL APO 40×/0.95, PL APO 63×/1.4 Oil DIC) and LSM-software ZEN 2 v10.0. The following emission bands were used: DAPI, 410–579 nm; FITC, 493–579 nm; TurboFP635, 582–754 nm; Alexa Fluor-594, 585–733 nm.

### 4.6. Software

Image editing and analysis were performed using PaintNET v5.0.8, Adobe Photoshop v21.2.9.67, InkScape v0.92.4 and Fiji v1.54f. The quality of raw fastq reads was assessed by FastQC v0.11.7. The alignment of fastq reads to the reference genome was done by Hisat v2.2.0.

## 5. Conclusions

This study adds new data on the genotype–phenotype interaction in EBS, which is important as the variants found are recurrent. It is important to emphasize the uniqueness of the clinical data obtained and the description of the patients obtained during the neonatal period of life. *POGLUT1* missense heterozygous mutation found in Proband B was reported to connect NOTCH pathway impairment with the development of autosomal dominant genodermatosis Dowling-Degos disease 4 (DDD4)—pathogenic conditions of skin with progressive and disfiguring reticulate hyperpigmentation.

## Figures and Tables

**Table 1:** EBS pathogenic variants associated with KRT14-associated pathology. Genome position (according to Varsome), missense mutation, reference single nucleotide polymorphism ID (rs), OMIM, Phenotype and MIM number are listed.

Gene	Position	Variant	rs	OMIM	Phenotype MIM Number	Phenotype	Gene/ Locus MIM Number
*Krt14*	Chr17:39742731 A>C	Met119Thr	rs28928893	EBS2F (131960)	131760	EBS severe with mottled pigmentation	148066
*KLHL24*	Chr3:119188886 A>G	Met1Val	rs886037956	EBS6 (617294)	131900 617294	EBS generalized intermediate with or without cardiomyopathy	611295
*POGLUT1*		Arg279Trp	rs58330629	615696 611295	615696	DDD4	615618
*Krt14*	Chrl7:.39742713 C>T	Arg125His	rs886037956	EBS1A	131760		148066

## Data Availability

Data contained within the article.
